# LRRK2 Kinase Inhibition Attenuates Astrocytic Activation in Response to Amyloid β_1-42_ Fibrils

**DOI:** 10.3390/biom13020307

**Published:** 2023-02-06

**Authors:** Alice Filippini, Valentina Salvi, Vincenzo Dattilo, Chiara Magri, Stefania Castrezzati, Robert Veerhuis, Daniela Bosisio, Massimo Gennarelli, Isabella Russo

**Affiliations:** 1 IRCCS Istituto Centro San Giovanni di Dio Fatebenefratelli, 25125 Brescia, Italy; 2 Oncology and Experimental Immunology Unit, Department of Molecular and Translational Medicine, University of Brescia, 25123 Brescia, Italy; 3 Biology and Genetics Unit, Department of Molecular and Translational Medicine, University of Brescia, 25123 Brescia, Italy; 4 Human Anatomy Unit, Department of Biomedical Sciences and Biotechnologies, University of Brescia, 25123 Brescia, Italy; 5 Amsterdam UMC, Psychiatry, Amsterdam Public Health Research Institute and Neuroscience Amsterdam, Vrije Universiteit Amsterdam, 1081 HV Amsterdam, The Netherlands; 6 Amsterdam UMC, Department of Clinical Chemistry, Vrije Universiteit Amsterdam, 1081 HV Amsterdam, The Netherlands

**Keywords:** LRRK2, Alzheimer’s disease, astrocytes, amyloid-β, neuroinflammation

## Abstract

Intracerebral accumulation of amyloid-β in the extracellular plaques of Alzheimer’s disease (AD) brains represents the main cause of reactive astrogliosis and neuroinflammatory response. Of relevance, leucine-rich repeat kinase 2 (LRRK2), a kinase linked to genetic and sporadic Parkinson’s disease (PD), has been identified as a positive mediator of neuroinflammation upon different inflammatory stimuli, however its pathogenicity in AD remains mainly unexplored. In this study, by using pharmacological inhibition of LRRK2 and murine primary astrocytes, we explored whether LRRK2 regulates astrocytic activation in response to amyloid-β_1-42_ (Aβ_1-42_). Our results showed that murine primary astrocytes become reactive and recruit serine 935 phosphorylated LRRK2 upon Aβ_1-42_ fibril exposure. Moreover, we found that pharmacological inhibition of LRRK2, with two different kinase inhibitors, can attenuate Aβ_1-42_-mediated inflammation and favor the clearance of Aβ_1-42_ fibrils in astrocytes. Overall, our findings report that LRRK2 kinase activity modulates astrocytic reactivity and functions in the presence of Aβ_1-42_ deposits and indicate that PD-linked LRRK2 might contribute to AD-related neuroinflammation and pathogenesis.

## 1. Introduction

Alzheimer’s disease (AD) is the most devastating neurodegenerative disease in the adult population and represents the most frequent cause of dementia worldwide [1]. AD is neuropathologically characterized by progressive neurodegeneration, together with deposition of aggregated proteins, extracellular amyloid-β and intracellular hyperphosphorylated Tau [1,2]. Although the mechanisms underlying AD onset and progression are not yet fully elucidated, it is well known that neuroinflammation plays a critical role in the pathology [3,4]. Neuroinflammation, driven by activation of microglia and astrocytes, is a defense mechanism that protects the brain against pathogens or other inflammatory stimuli, with beneficial effects on brain tissue repair [5]. However, a sustained inflammatory response can turn cytotoxic and lead to cellular and tissue damage, thus promoting neurodegeneration and disease progression [4,5,6]. In this regard, several studies have reported the involvement of chronic neuroinflammation and glia activation in AD [7,8,9]. Reactive microglia and astrocytes have been detected in proximity to amyloid-β plaques in post-mortem brains of AD patients and animal models [10,11,12]. In addition, elevated levels of pro-inflammatory mediators, such as interleukin-1β (IL-1β), tumor necrosis factor-α (TNF-α) and interleukin-6 (IL-6), have been detected in both experimental models and post-mortem brains with AD [3,13,14]. Moreover, it has been shown that pro-inflammatory cytokines might further potentiate the enzymatic activity of Tau kinases, increasing deposition of intracellular phosphorylated Tau [15], and of γ- and β-secretases, leading to amyloid-β accumulation [16,17]. Overall, these observations support a key contribution of the neuroinflammation on promoting AD pathogenesis.

Leucine-rich repeat kinase 2 (LRRK2), a protein linked to genetic and sporadic Parkinson’s disease (PD) [18,19,20], has been revealed as a positive mediator of neuroinflammation, both in in vitro and in vivo studies [21,22,23,24,25,26,27,28,29,30]. LRRK2 is a multimeric protein characterized by an enzymatic core with GTPase and serine/threonine kinase activities and several domains surrounding these enzymatic domains involved in the assembly of signaling complexes [31]. LRRK2 is a physiologically multiphosphorylated protein, with clusters of both heterologous phosphorylation and autophosphorylation sites [32]. In regard to heterologous phosphorylation sites, serine 910/935 residues have been shown to be constitutively phosphorylated by multiple kinases (PKA; IKKα and β and CK1α) involved in different cellular pathways [33,34] and widely used as a marker of LRRK2 pharmacological kinase inhibition both in vitro and in vivo [32,35,36].

As the pathogenicity of LRRK2-mediated inflammation in AD remains mainly unexplored, and given that neuroinflammation widely contributes to AD, here, we explored whether LRRK2 regulates astrocytic activation in response to amyloid-β_1-42_ (Aβ_1-42_). We found that murine primary astrocytes become reactive and recruit serine 935 phosphorylated LRRK2 upon Aβ_1-42_ fibril exposure. Of interest, our results showed that the inhibition of LRRK2 kinase activity, with two different inhibitors, attenuates the induction of pro-inflammatory IL-1β cytokine. Moreover, we observed that astrocytes treated with LRRK2 kinase inhibitors exhibited a strong reduction in the amount of intracellular amyloid-β fibrils, which is partly rescued by blocking lysosomal degradation, suggesting that LRRK2 is involved in the clearance of Aβ_1-42_ fibrils.

Overall, our findings indicated that LRRK2 kinase activity regulates amyloid-β-mediated astrocytic activation and functionality and, importantly, suggested that LRRK2 might contribute to AD-related neuroinflammation and pathogenesis.

## 2. Materials and Methods

### 2.1. Primary Astrocytes

C57BL/6J wild-type mice were maintained under a 12 h light–dark cycle at room temperature (RT, 22 °C) with ad libitum food and water. Animal procedures were performed in accordance with European Community Directive 2010/63/UE and approved by the Ethics Committee of the University of Brescia (Project ID: 800-2017). Moreover, the research protocol has been approved by the Ethics Committee of IRCCS San Giovanni di Dio–Fatebenefratelli (n° 90-2021 and Prot. 290/2021).

Primary astrocytes were derived from pups at post-natal days 2–4 (P2–P4). Briefly, cerebral cortices were dissociated in cold PBS; the cell suspension was maintained at RT for 5 min and the top fraction was centrifuged at 1000 rpm 5 min. The cells were then resuspended in astrocytes medium containing high-glucose DMEM (Immunological Sciences, Rome, Italy), 10% fetal bovine serum (FBS, ThermoFisher Scientific, Waltham, MA, USA), 2mM L-Glutamine (ThermoFisher Scientific) and penicillin/streptomycin (ThermoFisher Scientific). Cells obtained from five brains were then seeded in 175 cm^2^ flasks and maintained at 37 °C with 5% CO_2_. After 4 days, the medium was changed and then the cells were maintained in culture until confluence (DIV7-9), when the cells were processed for experimental applications. The purity of primary astrocytic culture was verified by immunostaining with CD11b and glial fibrillar acidic protein (GFAP) antibodies, microglia and astrocyte markers, respectively. In our cultures, the amount of microglia contaminants was ~13%.

### 2.2. Aβ_1-42_ Fibril Generation and Validation

Aβ_1-42_ fibrils were generated as previously reported [37,38,39]. Specifically, human Aβ_1-42_ (Bachem, Bubendorf, Switzerland) was dissolved in cold hexafluoroisopropanol (HFIP, Merck/Sigma-Aldrich, St. Louis, MO, USA) and maintained under rotation at RT overnight. The solution of Aβ_1-42_ was then aliquoted, speed-vacuum dried and stored at −80 °C until use. Before treatment, Aβ_1-42_ was dissolved in anhydrous dimethylsulfoxide (DMSO, Merck/Sigma-Aldrich) and sonicated 10 min at RT to remove possible peptide aggregation. Then, to obtain an enriched fibril preparation, Aβ_1-42_ was further resuspended in 10 mM HCl and incubated at 37 °C for 48 h. Instead, fluorescent FAM-labelled Aβ_1-42_ fibrils were prepared by dissolving dried un-labelled Aβ_1-42_ with 20 µM FAM-Aβ_1-42_ (FAM-Aβ, Anaspec Inc., Fremont, CA, USA) in anhydrous DMSO; then, the solution was sonicated 10 min at RT, diluted in 10 mM sterile HCl to obtain 100 µM FAM-Aβ_1-42_ and kept at 37 °C for 48 h.

The fibrillization of Aβ_1-42_ fibrils was verified by ThioflavinT (ThioT, Merck/Sigma Aldrich) assays and transmission electron microscopy (TEM). In brief, 7 µg of fibrils were incubated for 1 min at RT with 5 µM of ThioT. Control measurement was performed with 5 µM ThioT in HCl for detection of background fluorescence intensity. Fluorescence emission was recorded at 482 nm with excitation at 450 nm by using the PerkinElmer^®^ EnSight-Multimode Plate Reader. For TEM, Aβ_1-42_ 1 µM samples were incubated on a 400 mesh formvar-coated grid (TAAB Ltd., Berks, UK) for 2 min at RT. After removing the excess solution from the grid, samples were negatively stained with Uranyless (Electron Microscopy Sciences) for 2 min at RT and examined with a transmission electron microscope (Tecnai G2 Spirit; FEI Company, Eindhoven, The Netherlands) at 80 kV.

### 2.3. Reactive Oxygen Species (ROS) Detection

ROS generation was measured using the CellROX^®^ Green Reagent (C10444, ThermoFisher Scientific) following manufacturer’s instructions. Briefly, astrocytes were treated with 10 µM Aβ_1-42_ fibrils, or 10 mM-DMSO HCl as a control, for 16 h. Successively, astrocytes were washed once with 1X PBS and treated with 5 µM CellROX^®^ Green Reagent in cell medium for 30 min at 37 °C. After treatment, the cells were washed three times with 1X PBS and the ROS fluorescence was detected using PerkinElmer^®^ EnSight-Multimode Plate Reader, setting the excitation at 485 nm and the emission at 520 nm.

### 2.4. Compound and Cell Treatment

LRRK2 inhibitors were dissolved in DMSO. GSK2578215A (GSK, Tocris Bioscience, Bristol, UK) and IN-1 were used at 2 µM and 1 µM, respectively. Specifically, astrocytes were exposed to LRRK2 inhibitors 90 min before treatment with Aβ_1-42_ fibrils.

Chloroquine (CQ, Merck/Sigma Aldrich), an inhibitor of lysosomal activity, was dissolved in ultrapure distilled water and used on primary astrocytes at 25 µM for 16 h.

For Western blot and ROS detection experiments, astrocytes were treated with unlabeled 10 µM Aβ_1-42_ fibrils, or 10 mM-DMSO HCl as a control, for 16 h, while, for FACS experiments, astrocytes were treated with FAM-Aβ_1-42_ fibrils of 10 µM, or 10 mM-DMSO HCl as a control, for 2 h to analyze Aβ_1-42_ uptake. For the immunofluorescence, astrocytes were treated with 10 µM Aβ_1-42_ fibrils for 2 h.

During all the treatments, primary astrocytes were cultured in medium containing 1% FBS.

### 2.5. Cell Immunofluorescence and Imaging

Cells were washed once with PBS 1X and then fixed with paraformaldehyde (PFA) 4% pH 7.4 15 min at RT. After three washes with PBS 1X, permeabilization with PBS/Triton-X-100 0.3% was performed for 5 min at RT. Next, cells were saturated with blocking solution (FBS 5% in PBS/Triton-X-100 0.3%) for 1 h at RT and incubated with primary antibody anti-β amyloid clone 6E10 (Biolegend 803004, 1:50), anti-GFAP (Invitrogen 13-0300, 1:500) and anti-CD11b (BD Biosciences 550282, 1:100) diluted in blocking solution. After three washes in PBS 1X, cells were incubated with secondary antibody AlexaFluor 488 or 594 (ThermoFisher Scientific, 1:500) for 1 h at RT. After several washes in PBS 1X, cells were mounted using Prolong Gold Antifade reagent containing DAPI (ThermoFisher Scientific). Images were acquired with a Zeiss Axioplan2 fluorescence microscope with a 63× oil immersion objective (Carl Zeiss AG, Oberkocken, Germany).

### 2.6. Aβ_1-42_ Intracellular Quantification

Aβ_1-42_ intracellular amount was quantified by using different assays and approaches. For FAM-Aβ_1-42_ experiments, following incubation with FAM-Aβ_1-42_ fibrils, cells were washed once with 1X PBS, incubated with quenching solution (0.2% Trypan blue in 1X PBS pH 4.4) for 1 min at RT to avoid the detection of FAM-Aβ_1-42_ fibrils present or stuck on the cell membrane and then washed three times with 1X PBS. Astrocytes were then collected using 0.25% trypsin, centrifuged at 420× *g* 5 min at 4 °C and washed once with cold FACS Buffer (1X PBS, 1% FBS, 2 mM EDTA). After a further centrifugation, cells were resuspended in cold FACS Buffer and kept in ice until FACS analysis. Fluorescence emission was read at 521 nm on a MACSQuant flow cytometer (Miltenyi Biotec, Bergisch Gladbach, Germany) and analyzed with FlowJo software (Tree Star Inc., Ashland, OR, USA). At least 70,000 cells were analyzed for each sample. We assessed the median fluorescence intensity (MFI) of cells analyzed and the values of untreated cells represented the autofluorescence.

For the experiments related to lysosomal activity inhibition, primary astrocytes were exposed to 25 µM CQ and to 2 µM LRRK2 GSK inhibitor for 120 min and 90 min, respectively, before the treatment with Aβ_1-42_ fibrils. The cells were analyzed 16 h after the treatment with Aβ_1-42_ fibrils. After treatment, the cells were fixed and immunostained for GFAP and Aβ_1-42_ as described above. Quantification of intracellular Aβ_1-42_ was performed using ImageJ software, calculated as fluorescence intensity divided by cellular area (detected by GFAP) and expressed as fluorescence intensity/µm^2^. At least thirty cells were randomly chosen in a minimum of three independent experiments. Images were acquired using a Zeiss Axioplan2 microscope with a 63× oil-immersion objective (Carl Zeiss AG).

### 2.7. Cell Lysis and Western Blotting

Astrocytes were washed twice with PBS 1X, solubilized with cold lysis buffer (20 mM Tris–HCl pH 7.5, 150 mM NaCl, 1 mM EDTA, 2.5 mM sodium pyrophosphate, 1 mM β-glycerophosphate, 1 mM Na_3_VO_4_, 1% Triton-X-100, protease inhibitors), incubated on ice 20 min and centrifuged at 14,000 rpm at 4 °C. The supernatant was collected for protein electrophoresis. Specifically, total proteins were separated using 7.5% acrylamide sodium dodecyl sulphate (SDS)-PAGE gels. Subsequently, proteins were transferred on a polyvinylidene difluoride (PVDF) membrane (Bio-Rad, Hercules, CA, USA), saturated with non-fat dry milk 5% in TBS-Tween 1% (TBST) 1 h at RT and incubated with primary antibodies: anti-β amyloid clone 6E10 (Biolegend, 803004, 1:1000), anti-GAPDH (ThermoFisher Scientific MA5-15738, 1:30,000), anti-IL-1β (R&D System AF-401-NA, 1:2000), anti-LRRK2 phospho serine 935 (Abcam ab133450, 1:300), anti-LRRK2 (Abcam ab133474, MJFF2 1:300), anti-clusterin (R&D System AF2747, 1:2000). Next, membranes were incubated with horseradish peroxidase (HRP)-conjugated secondary antibodies (Merck/Sigma Aldrich) for 1 h at RT and then with ECL substrate of HRP.

### 2.8. Statistical Analysis

All data are expressed as mean *±* SEM and represent at least three sets of experiments. Statistical significance of differences between two groups was assessed by unpaired *t*-test, while multiple comparison was measured using one-Way ANOVA followed by Tukey’s post hoc test. Cumulative frequency distributions were compared with a Kolmogorov–Smirnov test. Data were analyzed using Prism software (GraphPad) and statistical significance was taken at *p* < 0.05.

## 3. Results

### 3.1. Astrocytic Activation in Response to Aβ_1-42_ Fibrils Priming

**Figure 1:**
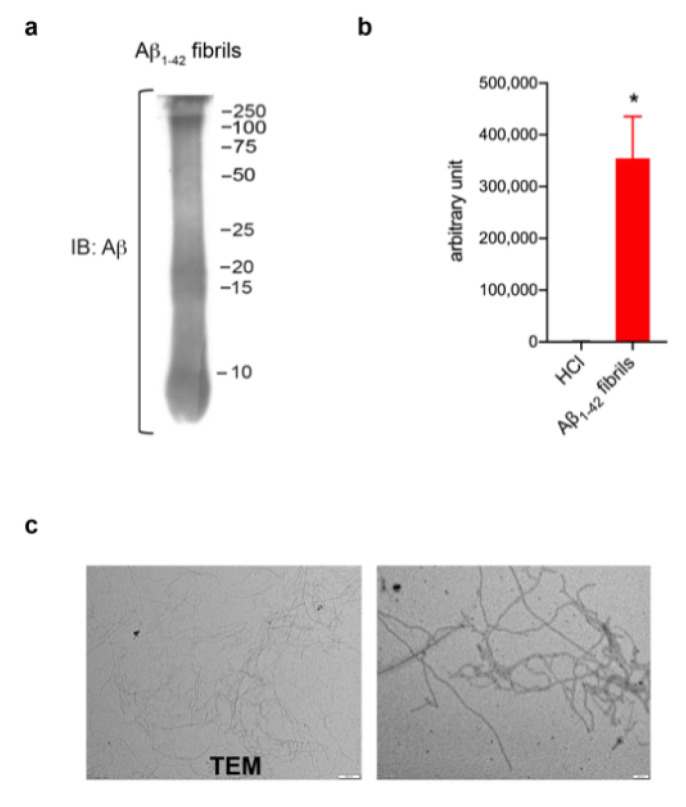
Aβ_1-42_ fibril generation and validation. (**a**) Immunoblotting detecting Aβ_1-42_ fibrils using anti-β amyloid clone 6E10 antibody. (**b**) ThioT assay shows a greater amount of fluorescent signal in fibril-enriched preparation compared to control solvent DMSO-HCl. Data are representative of three independent preparations and are expressed as mean ± SEM. Data were analyzed using unpaired *t*-test, * *p* = 0.0120. (**c**) TEM performed on Aβ_1-42_ preparation reveals thread-like fibril structure. Scale bar 500 nm and 200 nm.

In order to investigate a potential role of LRRK2 in astrocytic activation upon AD-related aggregates, we first generated and validated Aβ_1-42_ fibrils (Figure 1). Aβ_1-42_ fibrils were prepared from Aβ_1-42_ monomeric protein incubated for 48 h to induce aggregation. The formation of fibrils was verified using several approaches. Specifically, Western blot confirmed the presence of a smear with large Aβ aggregates with high molecular weight (Figure 1a), ThioT assay detected a greater amount of fluorescence signal in fibrils preparation compared to control solvent (Figure 1b) and TEM examination reported thread-like fibrillar structures (Figure 1c). Taken together, these results indicate the high quality of our Aβ_1-42_ fibril-enriched preparation.

**Figure 2:**
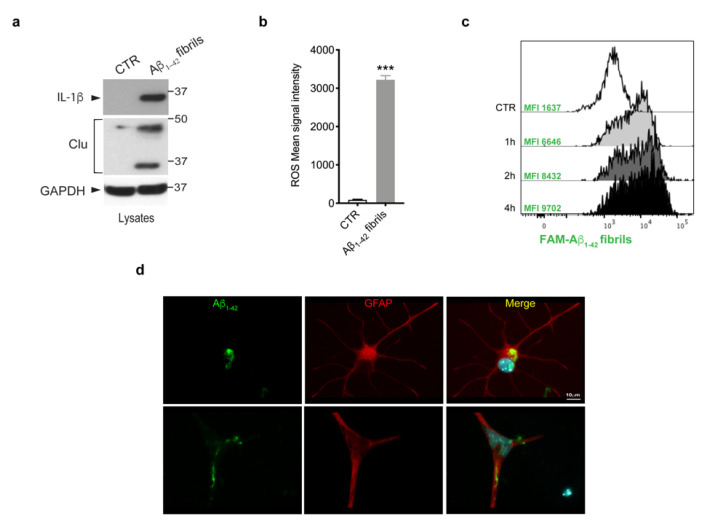
Astrocytes activation upon Aβ_1-42_ fibrils. (**a**) Cell lysates of primary astrocytes treated with 10 μM Aβ_1-42_ fibrils for 16 h, or with 10 mM-DMSO HCl as a control, were subjected to immunoblotting using IL-1β, clusterin (Clu) and GAPDH antibodies. Data are representative of several independent experiments (IL-1β: *n* = 10, clusterin: *n* = 4). (**b**) ROS mean signal intensity was assessed in primary astrocytes exposed to 10 μM Aβ_1-42_ fibrils for 16 h or to 10 mM-DMSO HCl as a control. Data are representative of five independent experiments and are expressed as mean ± SEM. Data were analyzed using unpaired *t*-test, *** *p* < 0.0001. (**c**) Representative cytofluorimetric profile and MFI values of astrocytes exposed to 10 µM FAM-Aβ fibrils for different time points (1 h, 2 h and 4 h). The control curve represents the autofluorescence level of untreated cells. (**d**) Representative images of astrocytes exposed to Aβ_1-42_ fibrils for 2 h, fixed and immunostained for Aβ_1-42_ (green) and GFAP (red). Nuclei were stained with DAPI (blue). Scale bar 10 µm.

Given that multiple lines of evidence have indicated that amyloid-β deposits trigger glia activation and inflammation [3,40], we investigated whether Aβ _1-42_ fibrils were able to activate primary astrocytes. To this purpose, we treated murine primary astrocytes with Aβ_1-42_ fibrils, or with DMSO-HCl as a control, and we assessed the induction of IL-1β cytokine, one of the most crucial pro-inflammatory cytokines in AD [41,42,43,44]; of clusterin, a stress-related protein [45]; and the generation of ROS. As shown in Figure 2a,b, we observed a significant increase of all the inflammatory markers analyzed, indicating that Aβ_1-42_ fibrils triggered astrocytes toward a pro-inflammatory and reactive phenotype.

Next, we asked whether astrocytes were able to internalize Aβ_1-42_ fibrils. We first performed a time-course experiment in which cells were exposed to FAM-Aβ_1-42_ fibrils for 1 h, 2 h and 4 h and we evaluated fibril uptake through flow cytometry. As shown by FACS analysis of MFI (Figure 2c), the uptake of Aβ_1-42_ fibrils was clearly detectable after 1 h of treatment and further increased at longer time points. Moreover, we observed the ability of astrocytes to internalize Aβ_1-42_ fibrils, even on fixed cells, via imaging, as reported in the Figure 2d. Taken together, these results indicate that the treatment with Aβ_1-42_ fibrils triggered astrocytes toward a reactive phenotype with the ability to internalize fibrils.

### 3.2. LRRK2 Kinase Inhibition Attenuates Aβ_1-42_ Fibril-Mediated Astrocytic Activation

**Figure 3:**
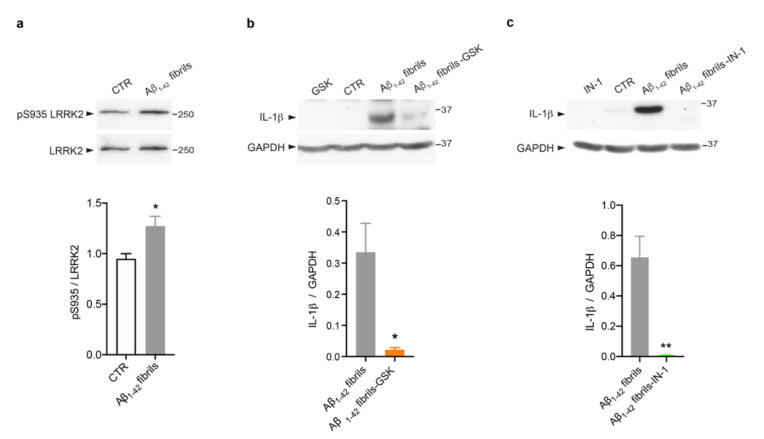
LRRK2 kinase activity controls Aβ_1-42_ fibril-mediated astrocytic inflammation. (**a**) Cell lysates of primary astrocytes treated for 16 h with Aβ_1-42_ fibrils, or with DMSO-HCl 10 mM- as control, were subjected to immunoblotting using LRRK2-phosphoserine 935 (pS935) and LRRK2 antibodies. Data are representative of three independent experiments and are expressed as the mean ± SEM. Data were analyzed using unpaired *t*-test, * *p* = 0.0423. (**b**) Cell lysates of primary astrocytes treated with Aβ_1-42_ fibrils, Aβ_1-42_ fibrils and GSK, GSK alone or DMSO as control were subjected to immunoblotting using IL-1β and GAPDH antibodies. Data are representative of three independent experiments and are expressed as the mean ± SEM. Data were analyzed using unpaired *t*-test, * *p* = 0.0282. (**c**) Cell lysates of primary astrocytes treated for 16 h with Aβ_1-42_ fibrils, Aβ_1-42_ fibrils and IN-1, IN-1 alone or DMSO as control were subjected to immunoblotting using IL-1β and GAPDH antibodies. Data are representative of three independent experiments and are expressed as the mean ± SEM. Data were analyzed using unpaired *t*-test, ** *p* = 0.0099.

Several studies, including some from our group, have revealed that LRRK2 kinase activity is crucial to mediate glia activation in response to different stimuli, such as LPS [23,24,25], HIV-1 Tat protein [46], manganese [47] and α-synuclein fibrils [22,23,27]. However, evidence linking LRRK2 to AD-related inflammation is still missing. Thus, we explored the possibility that LRRK2 is involved in astrocytic inflammation mediated by Aβ_1-42_ fibrils. We first investigated whether LRRK2 was activated upon Aβ_1-42_ fibril exposure. To this aim, we analyzed the phosphorylation of LRRK2 at serine 935 (pS935), which has been reported to be increased in microglia upon exposure to inflammatory stimuli [22,46,48,49]. Of interest, we observed that Aβ_1-42_ fibrils triggered an increment of pSer935 -LRRK2 compared to control astrocytes (Figure 3a), suggesting that LRRK2 is involved in the cellular pathways activated by Aβ_1-42_ fibrils and that is phosphorylated/activated upon being challenged, even in astrocytes, as observed in microglia.

Then, we assessed whether LRRK2 kinase activity controls the induction of astrocytic inflammation. Thus, we treated cells with Aβ_1-42_ fibrils for 16 h in the presence of LRRK2 kinase inhibition. To this aim, we used two different compounds, GSK [50] and IN-1 [51]. Of note, both LRRK2 inhibitors attenuate the inflammatory response mediated by Aβ_1-42_ fibrils priming, as revealed by the strong reduction of IL-1β (Figure 3b,c). Taken together, these results indicate that LRRK2 kinase activity is a regulator of Aβ_1-42_ fibril-mediated inflammation in astrocytes.

### 3.3. Astrocytes with LRRK2 Kinase Inhibition Exhibited Increased Clearance of Aβ_1-42_ Fibrils

**Figure 4:**
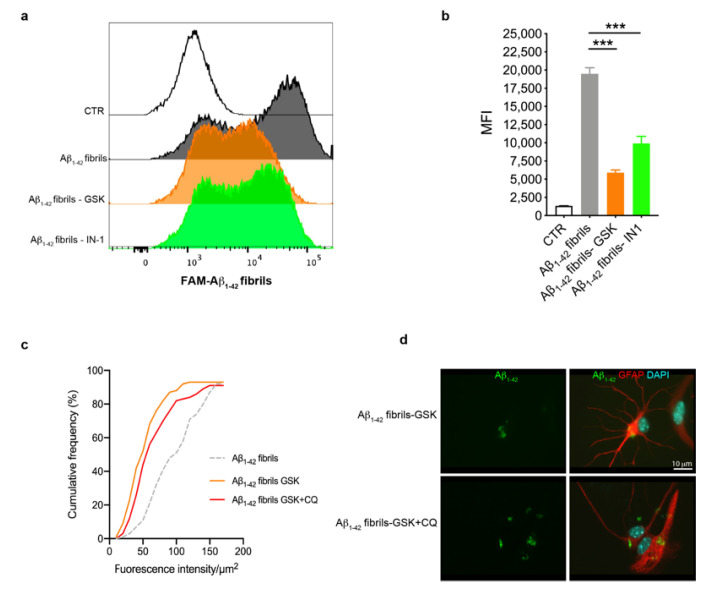
Astrocytes with LRRK2 kinase inhibition exhibited increased clearance of FAM-Aβ_1-42_ fibrils. (**a**) Astrocytes were treated with 2 μM GSK or 1 μM IN-1 for 90 min before the treatment with 10 μM FAM-Aβ_1-42_ fibrils for 2 h. The representative cytofluorimetric profile of the intracellular FAM-Aβ_1-42_ signal in the different groups was analyzed. The CTR curve represents the autofluorescence level of untreated cells. (**b**) Median fluorescence intensity (MFI) of cells analyzed by FACS is shown as mean ± SEM; the CTR histogram represents the autofluorescence level of untreated cells. Data were analyzed using one-way ANOVA with Tukey’s post hoc test; *** *p* < 0.0001. (**c**) Cumulative frequency distributions of intracellular Aβ_1-42_ fluorescence (Aβ_1-42_ fibrils = 92 cells, Aβ_1-42_ fibrils GSK = 93 cells and Aβ_1-42_ fibrils GSK + CQ = 91 cells). Cumulative frequency distributions of Aβ_1-42_ fibrils GSK and Aβ_1-42_ fibrils GSK + CQ samples were compared with Kolmogorov–Smirnov test, *p* = 0.0364. Quantification of intracellular Aβ_1-42_ fibrils was calculated as fluorescence intensity/μm^2^ from three independent experiments (~30 cells per experiment). (**d**) Representative fluorescence microscopy images of astrocytes exposed to CQ and LRRK2 GSK inhibitor, or LRRK2 GSK inhibitor alone, before treatment with Aβ_1-42_ fibrils for 16 h, fixed and immunostained for Aβ_1-42_ (green) and GFAP (red). Nuclei were stained with DAPI (blue). bar 10μm.

Previous studies have shown that LRRK2 affects the phagocytic functions of glia upon inflammatory priming [46,52,53]. Starting from these observations, we explored whether LRRK2 kinase activity was involved in the uptake of Aβ_1-42_ fibrils by astrocytes through FAM-Aβ_1-42_ fibrils and flow cytometry. To this aim, we treated astrocytes with LRRK2 inhibitors (GSK or IN-1) or, as control, DMSO-containing medium, for 90 min before exposing cells to FAM-Aβ_1-42_ fibrils for 2 h, after which we performed FACS analysis. By analyzing the intracellular FAM-Aβ_1-42_ signal of cells as MFI, we found that astrocytes treated with LRRK2 inhibitors exhibited a strong reduction of the MFI compared to cells treated with FAM-Aβ_1-42_ fibrils alone (Figure 4a,b), indicating that astrocytes with LRRK2 inhibition exhibited a decreased amount of intracellular Aβ_1-42_ fibrils. To investigate if the reduced MFI was mediated by the effect of LRRK2 kinase activity on the degradation rather than the uptake of FAM-Aβ_1-42_ fibrils, we quantified the intracellular Aβ_1-42_ after blocking lysosomal degradation with CQ. To this aim, we treated cells with GSK alone or in combination with CQ before the treatment with Aβ_1-42_ fibrils for 16 h and we quantified the intracellular Aβ_1-42_ fluorescence signal after fixation. Of interest, the cumulative frequency distribution analysis revealed that astrocytes treated with CQ display Aβ_1-42_ fluorescence signal consistently shifted toward higher intensity values with respect to cells treated with LRRK2 GSK inhibitor alone (Figure 4c,d), showing a homogenous increase of intracellular Aβ_1-42_ in the presence of CQ. Taken together, these results indicate that LRRK2 might be involved in the degradation process of Aβ_1-42_ fibrils and, importantly, suggest that the kinase inhibition of LRRK2 may favor the clearance of Aβ_1-42_ fibrils in astrocytes.

## 4. Discussion

Chronic neuroinflammation widely contributes to neurodegeneration and progression of several neurodegenerative diseases, including AD [13]. In this regard, amyloid-β accumulates in the form of amyloid plaques in the CNS and represents the main cause of neuroinflammation [3,5,54,55]. Of interest, in the last decade LRRK2 has been identified as a key regulator of the neuroinflammatory response in microglia and astrocytes upon inflammatory priming [21,26,56]. However, studies exploring the role of LRRK2 in amyloid-β-related inflammation are still missing. In this work, by using LRRK2 kinase inhibition, we found that LRRK2 modulates astrocyte activation upon exposure to AD-related amyloid deposits. Specifically, we established for the first time that the inhibition of LRRK2 kinase activity attenuates the induction of IL-1β cytokine and favors the clearance of Aβ_1-42_ fibrils, suggesting that LRRK2-related astrocytic functions might contribute to AD pathology.

Neuroinflammation represents a well-described event in AD and it is now emerging as one of the leading causes of the pathology [6,13,57,58,59]. Astrogliosis has been detected in the brains of AD patients [60] and of animal models [61,62]. In addition, several studies have shown that extracellular amyloid-β deposits trigger glial activation and inflammatory response [3,63]. Furthermore, glia reactivity and neuroinflammation have been reported to cause amyloid-β and tau protein aggregation, which are detrimental to neuronal and synaptic health [14]. Overall, these observations indicate that neuroinflammation is involved in different aspects of AD pathology and plays a crucial role in the progression of the disease. In this scenario, LRRK2, which is a positive mediator of neuroinflammation, could mediate and contribute to AD pathogenesis. To test this hypothesis, we asked whether LRRK2 can be activated and modulate astrocytic reactivity upon Aβ_1-42_ fibrils exposure. To assess inflammatory response in our experiments, we analyzed IL-1β induction, which is one of the most relevant pro-inflammatory cytokines, reported to be crucial for AD neuroinflammation and pathogenesis [3,4,64]. As expected, we found that Aβ_1-42_ fibrils mediate astrocytic activation, as detected by the strong generation of IL-1β, clusterin and ROS generation. Interestingly, for the first time, we observed that LRRK2 is activated upon Aβ_1-42_ fibril priming, as shown by the increased phosphorylation levels of LRRK2 Ser935, thus indicating that LRRK2 is actively involved in astrocytic pathways triggered by amyloid-β. In accord with these findings, our research and that of other groups have shown increased levels of phosphorylated LRRK2 in microglia upon different inflammatory stimuli, which correlated to the production of pro-inflammatory cytokines [22,46,48,49,65].

Of note, given that LRRK2 has been robustly associated to the modulation of inflammatory response [21,26,66], we then investigated whether LRRK2 kinase activity was implicated in the amyloid-β -mediated astrocytic inflammation. Our results showed that astrocytes with LRRK2 kinase inhibition exhibit a strong reduction of IL-1β generation after Aβ_1-42_ fibril exposure. These observations indicate that LRRK2 kinase activity regulates inflammation mediated by amyloid-β fibrils and that, as well as in microglia, LRRK2 might be a common modulator of the astrocytic inflammatory response. In support to this hypothesis, Munoz and colleagues reported that LRRK2 IN-1 inhibitor attenuated IL-6 cytokine secretion upon IL-1β priming in human primary astrocytes [28]. This is in accord with what we found in microglial cells, which exhibited reduced levels of IL-1β and cyclooxygenase-2 upon LPS priming when treated with LRRK2 inhibitors [23]. LRRK2 was found to control the induction of inflammation through protein kinase A (PKA)-mediated phosphorylation of nuclear factor kappa-B (NF-κB) inhibitory subunit p50 [23]. Our findings, that kinase inhibition of LRRK2 also exhibits a strong attenuation of IL-1β production in astrocytes upon exposure to Aβ_1-42_ fibrils, suggest that, also in astrocytes, the modulatory effects of LRRK2 on inflammatory response to amyloid-β fibrils may involve the PKA-NFκB pathway.

Microglia and astrocytes, efficient scavengers of the brain [67], have been found to be closely associated with amyloid plaques in AD brains and their involvement in amyloid-β clearance has gained increasing interest over the years [38,68,69]. In this context, AD pathology is hypothesized to be caused by an imbalance between amyloid-β production and clearance that leads to amyloid-β accumulation in the brain [70], thus supporting the importance of glia functions in the disease. Interestingly, LRRK2, through its kinase activity, has been shown to affect also phagocytosis and clearance of amyloid proteins by glial cells [46,52,71]. Specifically, it has been demonstrated that microglia with LRRK2 genetic deletion exhibited increased clearance of a-synuclein [53] and astrocytes with an enhanced LRRK2 kinase activity (LRRK2 pathological mutations) displayed reduced degradative capacity [52,72,73]. Starting from these premises, we then investigated whether LRRK2 was involved in the uptake/clearance of amyloid-β fibrils. Interestingly, our results revealed that the inhibition of LRRK2 kinase activity increases the ability of astrocytes to degrade Aβ_1-42_ fibrils. This is an important observation as it has been shown that astrocytes can engulf large amounts of amyloid-β but fail to properly digest the amyloid-β, which then accumulates and eventually leads to severe lysosomal and cellular dysfunctions [74]. Engulfment of Aβ_1-42_ by astrocytes may initially be a protective clearance mechanism, but an overburden can clearly be detrimental for astrocytic functions, leading, with time, to cell dysfunction, Aβ_1-42_ secretion and progression of AD [74]. Taken together, our findings indicate that LRRK2 is involved in the endo-lysosome pathway that leads to Aβ_1-42_ fibril degradation and, importantly, suggest that LRRK2 kinase inhibition might enhance the ability of astrocytes to clear amyloid-β and to preserve cell physiology.

## 5. Conclusions

Neuroinflammation strongly contributes to AD pathophysiology. Using LRRK2 kinase inhibition, we found that LRRK2 modulates the activation of astrocytes upon exposure to Aβ_1-42_ fibrils in vitro. Whereas the inhibition of LRRK2 kinase activity strongly attenuated the induction of IL-1β, it favored the clearance of Aβ_1-42_ fibrils by astrocytes. Overall, our study showed that LRRK2 kinase activity controls astrocyte reactivity induced by Aβ_1-42_ fibrils and indicates that LRRK2 might be involved in AD-related neuroinflammation and pathogenesis. However, future studies performed on transgenic mouse models of AD and on the post-mortem brain of AD patients might shed more light in the implication of PD-linked LRRK2 to AD pathogenesis and could contribute to dissecting novel pathological mechanisms for pharmacological intervention to limit AD progression.

## Data Availability

The datasets supporting the conclusion of this article are available in the ZENODO repository (10.5281/zenodo.7463238) from the corresponding author upon reasonable request.

## Abbreviations

AD	Alzheimer’s disease
LRRK2	leucine-rich repeat kinase 2
PD	Parkinson’s disease
GSK	GSK2578215A
IN-1	inhibitor-1
α-syn	α-synuclein
NF-κB	nuclear factor kappa-B
LPS	lipopolysaccharide
COX-2	cyclooxygenase -2
IL-1β	interleukin-1β
TNF- α	tumor necrosis factor-α
IL-6	interleukin-6
DMEM	Dulbecco’s Modified Eagle Medium
FBS	fetal bovine serum
GFAP	glial fibrillar acidic protein
HFIP	hexafluoroisopropanol
DMSO	dimethylsulfoxide
ThioT	thioflavin T
TEM	transmission electron microscopy
ROS	reactive oxygen species
FACS	fluorescence-activated cell sorting
PBS	phosphate-buffered saline
RT	room temperature
PFA	paraformaldehyde
CQ	chloroquine
SDS	sodium dodecyl sulphate
PVDF	polyvinylidene
TBST	TBS-tween
GAPDH	glyceraldehyde-3-phosphate dehydrogenase
HRP	horseradish peroxidase
EDTA	ethylenediaminetetraacetic acid
MFI	median fluorescence intensity
LPS	lipopolysaccharide
PKA	protein kinase A
